# Calcium/Calmodulin-Dependent Protein Kinase II Inhibitors Mitigate High-Fat Diet–Induced Obesity in Mice

**DOI:** 10.1155/jobe/5530467

**Published:** 2025-06-30

**Authors:** Naoyuki Kawao, Ryosuke Satoh, Yuya Mizukami, Katsumi Okumoto, Genzoh Tanabe, Osamu Muraoka, Reiko Sugiura, Hiroshi Kaji

**Affiliations:** ^1^Department of Physiology and Regenerative Medicine, Kindai University Faculty of Medicine, Osakasayama, Osaka, Japan; ^2^Laboratory of Molecular Pharmacogenomics, Department of Pharmaceutical Sciences, Faculty of Pharmacy, Kindai University, Higashi-Osaka, Osaka, Japan; ^3^Life Science Research Institute, Kindai University, Osakasayama, Osaka, Japan; ^4^Laboratory of Organic Chemistry, Department of Pharmacy, Faculty of Pharmacy, Kindai University, Higashi-Osaka, Osaka, Japan; ^5^Pharmaceutical Research and Technology Institute, Kindai University, Higashi-Osaka, Osaka, Japan

**Keywords:** adipocytes, adipose tissues, CaMKII, obesity

## Abstract

Calcium signaling contributes to obesity and its related disorders, such as diabetes. We herein investigated the effects of calcium/calmodulin-dependent protein kinase II (CaMKII) inhibitors on diet-induced obesity in mice. In mice fed a high-fat diet (HFD), the administration of the CaMKII inhibitor KN-93 and the glycolipid acremomannolipin A with the suppression of CaMKII phosphorylation reduced fat mass in the whole body, epididymal and subcutaneous white adipose tissue weights, and lipid accumulation in epididymal and subcutaneous white adipose tissues, but not muscle mass or bone mineral density at the tibia. Moreover, the administration of KN-93 and acremomannolipin A improved glucose intolerance in HFD-fed mice. In an in vitro study on preadipocytic 3T3-L1 cells and mouse adipose tissue-derived stromal cells, KN-93 and acremomannolipin A suppressed adipogenic differentiation, proliferation, and lipid accumulation. In conclusion, this is the first study to demonstrate that CaMKII inhibitors mitigated the development of diet-induced obesity in mice partly through the suppression of adipogenic differentiation, cell proliferation, and lipid accumulation in adipocytes. Inhibiting CaMKII could be a potential strategy for obesity treatment.

## 1. Introduction

Obesity is associated with various disorders, such as diabetes, hyperlipidemia, hypertension, cardiovascular disease, and nonalcoholic liver steatohepatitis [1, 2]. Although bariatric surgery was recently shown to be useful for attenuating most obesity-associated disorders [3], nonsurgical therapies are clinically demanded for the long-term management of patients with obesity. Anorexigenic drugs, such as mazindol, have been clinically used to treat severe obesity, but are addictive and drug resistance may develop through their effects on the central nervous system [4, 5]. Orlistat, naltrexone/bupropion, phentermine/topiramate, liraglutide, and semaglutide have been approved by the US Food and Drug Administration as antiobesity medications [3]. Liraglutide and semaglutide, which are long-acting glucagon-like peptide-1 receptor agonists, reduce body weight by suppressing energy intake [6, 7]. However, these antiobesity medications are not as effective as bariatric surgery, which limits their clinical dissemination. Therefore, the further development of novel drugs is required for improvements in the management of patients with obesity.

Excess energy is stored in white adipose tissue (WAT) in the body, which ultimately results in obesity [8]. Obesity is facilitated by the increased proliferation of adipocyte lineage cells, adipogenic differentiation, followed by elevated peroxisome proliferator-activated receptor γ (PPARγ) and aP2 levels, the formation of lipid droplets, and impaired lipolysis [9, 10]. Previous studies reported roles for calcium signaling in metabolic disturbances [11, 12]. Segal et al. demonstrated that higher intracellular calcium levels in adipocytes were partly involved in the development of insulin resistance in obese patients with Type 2 diabetes [11]. Guney et al. showed that the disruption of inositol trisphosphate receptors in adipocytes suppressed adipose tissue inflammation and insulin resistance in diet-induced obese mice [12]. Collectively, these findings suggest that calcium signaling contributes to obesity-related disorders. Regarding the involvement of the calcium-sensing enzyme calcium/calmodulin-dependent protein kinase II (CaMKII) in obesity, CaMKII was shown to contribute to glucose metabolism abnormalities in the livers of obese mice [13, 14]. The thromboxane A_2_/thromboxane A_2_ receptor axis was recently found to facilitate hepatic insulin resistance through CaMKII in obese mice [15]. Moreover, Dai et al. demonstrated that an adipocyte-specific CaMKII deficiency attenuated obesity-associated glucose intolerance and insulin resistance by enhancing insulin signaling in mice [16]. An in vitro study indicated that CaMKII signaling was related to adipogenic differentiation in mouse adipogenic 3T3-L1 cells [17]. Salagre et al. also revealed that a chronic melatonin treatment increased the phosphorylation of CaMKII in skeletal muscle and attenuated obesity in obese rats [18]. Taken together, these findings indicate the potential of calcium/CaMKII signaling as a drug target for obesity and its related disorders, including diabetes. However, the efficacy of CaMKII inhibitors in the treatment of obesity in vivo has not been fully studied.

KN-93 is the most widely used CaMKII inhibitor in in vivo and in vitro experiments [19]. It inhibits the activation of CaMKII by binding to calcium/calmodulin [20]. Lo et al. revealed that KN-93 prevented arrhythmic activity induced by catecholamines in rabbit pulmonary veins [21]. Wang et al. showed that KN-93 reduced elevated blood glucose levels in human prostaglandin F_2α_ receptor transgenic mice [22]. We previously isolated the glycolipid acremomannolipin A from the filamentous fungus *Acremonium strictum* as a potential calcium signal modulator [23]. Acremomannolipin A enabled calcineurin gene-deleted fission yeast to grow in the presence of chloride [23]. Moreover, acremomannolipin A inhibited CaMKII/IV activity in a preliminary in vitro kinase assay. Therefore, we herein investigated the effects of KN-93 and acremomannolipin A on obesity in high-fat diet (HFD)–induced obese mice.

## 2. Materials and Methods

### 2.1. Animals

Male C57BL/6J mice were obtained from CLEA Japan (Tokyo, Japan). Animal experiments were performed according to the guidelines of the National Institutes of Health and institutional rules for the use and care of laboratory animals at Kindai University. All procedures were approved by the Experimental Animal Welfare Committee of Kindai University (Permit number: KAME-27–029).

### 2.2. Animal Experiments

Five-week-old male mice were fed *ad libitum* with a normal diet (ND) or HFD (57% of calories from fat, CLEA Japan) for 8 weeks. Food intake was measured every 3 days. Mice were randomly divided into six groups: ND/control (*n* = 8), ND/KN-93 (*n* = 8), ND/acremomannolipin A (*n* = 8), HFD/control (*n* = 8), HFD/KN-93 (*n* = 8), and HFD/acremomannolipin A (*n* = 8) groups. In animal experiments, a total of 48 mice were used and each experimental group included eight mice. KN-93 (Merck Millipore, Billerica, MA, USA) and acremomannolipin A were dissolved in phosphate-buffered saline containing 1% dimethyl sulfoxide and administered to 5-week-old mice at intraperitoneal doses of 5.33 and 15 μmol/kg, respectively, every other day for 8 weeks. The doses of KN-93 and acremomannolipin A were selected based on previous studies and preliminary experiments [22, 24]. Twenty-four hours after their final administration, mice were euthanized with excess isoflurane (5%, Wako Pure Chemicals, Osaka, Japan) and tissue samples were collected and weighted.

### 2.3. Quantitative Computed Tomography (QCT) Analysis

A QCT analysis was performed using an X-ray CT system (LaTheta LCT-200; Hitachi Aloka Medical, Tokyo, Japan) according to the guidelines of the American Society for Bone and Mineral Research [25], as previously described [26]. After mice were anesthetized with 2% isoflurane, they were scanned using the following parameters: tube voltage 50 kVp, tube current 500 μA, axial field of view 48 mm, and voxel size of 48 × 48 × 192 μm, to analyze fat and muscle mass in the whole body as well as an isotropic voxel size of 24 μm to assess bone mineral density (BMD) at the tibia. To assess fat and muscle in the whole body, the region of interest was defined as the whole body. To evaluate total bone mineral content (BMC) and trabecular BMD at the tibia, the region of interest was defined as 1680-μm segments from 96 μm distal to the end of the proximal growth plate toward the diaphysis (70 slices). To measure cortical BMD at the tibia, the region of interest was defined as 2160-μm segments of the mid-diaphysis of the tibia (90 slices).

### 2.4. Grip Strength

Grip strength was analyzed using a grip strength meter (1027SM, Columbus Instruments, Columbus, OH, USA), as previously described [27]. After a mouse grasped a pull bar attached to a grip strength meter, its tail was continuously pulled until it released the pull bar. Grip strength was measured 5 times. The results obtained represent the average of each mouse.

### 2.5. Analyses of Hematological Parameters

Mice were euthanized with excess isoflurane (5%). Blood samples were collected by cardiac puncture and erythrocyte, leukocyte, and platelet numbers, and the hemoglobin level and hematocrit were examined using an automated hematology analyzer (XT-1800i, Sysmex, Kobe, Japan). Plasma creatinine levels were analyzed using LabAssay creatinine (Wako Pure Chemicals). Plasma aspartate aminotransferase (AST) and alanine aminotransferase (ALT) levels were measured using the Transaminase CII-test Wako (Wako Pure Chemicals). Plasma insulin levels were assessed using a mouse insulin enzyme-linked immunosorbent assay (Cat. No. AKRIN-011T, FUJIFILM Wako Shibayagi, Gunma, Japan).

### 2.6. Histological Analysis

After mice were euthanized with excess isoflurane (5%), epididymal and subcutaneous WAT was collected, fixed with 4% paraformaldehyde in phosphate buffer (pH 7.4) for 24 h, and then embedded in paraffin. Four-micrometer-thick sections were stained with hematoxylin and eosin. Hematoxylin and eosin-stained sections were photographed under a microscope (BZ-X810; Keyence, Osaka, Japan), and cross-sectional areas of lipid droplets were quantified using ImageJ (https://imagej.nih.gov/ij/) in a blinded manner.

### 2.7. Glucose Tolerance Test

A glucose tolerance test was performed as previously described [28]. Glucose (Wako Pure Chemicals) at 1.5 g/kg was administered intraperitoneally to mice. Glucose levels in blood samples obtained from the tail vein were measured using a glucometer (Glutest Ace; Sanwa Kagaku, Nagoya, Japan) before and 30, 60, 90, and 120 min after the injection.

### 2.8. Analysis of Triglyceride Levels in the Liver

Triglyceride levels in the liver were measured as previously described [29]. After mice were euthanized with excess isoflurane (5%), liver tissues were collected and homogenized in Milli-Q water, and lipids were extracted by the Folch method with some modifications. Triglyceride levels were analyzed using the LabAssay triglyceride kit (Wako Pure Chemicals). Quantified values are expressed as triglyceride weight per tissue weight of the liver (mg/g).

### 2.9. Cell Culture

Mouse preadipocytic 3T3-L1 cells were cultured in Dulbecco's modified Eagle's medium (DMEM) with 10% fetal bovine serum (FBS) and 1% penicillin/streptomycin. Adipose tissue-derived stromal cells (ADSCs) were obtained from the subcutaneous WAT of male C57BL/6J mice as previously described [30, 31]. Briefly, after mice were euthanized with excess isoflurane (5%), subcutaneous WAT was removed and digested with 2 mg/mL of collagenase (Gibco, Thermo Fisher Scientific, Osaka, Japan) at 37°C for 1 h. Digested tissues were centrifuged and filtrated through a cell strainer with 100 μm. We previously demonstrated using flow cytometry that ADSCs obtained from the subcutaneous adipose tissues of mice expressed positive mesenchymal stem cell surface markers (CD29, CD44, CD90, CD105, and Sca-1), but not negative markers (CD34, CD45, CD3, CD11b, Gr1, and TER-119) [31]. ADSCs were grown in DMEM containing 10% FBS and 1% penicillin/streptomycin. Mouse ADSCs from the second passage were used in experiments. 3T3-L1 cells and ADSCs from the second passage were seeded at 5.0 × 10^3^ cells/cm^2^ and cultured in DMEM containing 10% FBS, 10 μg/mL insulin, 1 nM dexamethasone, and 0.5 mM 3-isobutyl-1-methylxanthine to induce adipogenic differentiation for 6 days for real-time polymerase chain reaction (PCR) analyses or 12 days for Oil Red O staining.

### 2.10. Western Blotting

A Western blot analysis was performed as previously described [32]. Briefly, epididymal WAT and cells were lysed in a cell lysis buffer (Cell Signaling Technology, Danvers, MA, USA) containing protease and phosphatase inhibitors. After protein levels were measured using a bicinchoninic acid assay reagent (Pierce, Rockford, IL, USA), 4-μg protein aliquots were electrophoresed on 4%–20% Mini-Protean Tris-Glycine extended precast gels (BioRad Laboratories, Hercules, CA, USA). Separated proteins were transferred to a polyvinyl difluoride membrane (Millipore, Bedford, MA, USA). The membrane was incubated with a blocking buffer containing 3% skim milk for 1 h and then with an anti-CaMKII antibody (Cat. No. 4436, Cell Signaling Technology) diluted 1:1000, anti-phosphorylated CaMKII antibody (Cat. No. 12716, Cell Signaling Technology) diluted 1:1000, or an anti-β-actin antibody (Cat. No. 4970, Cell Signaling Technology) diluted 1:5000 at 4°C overnight. After the membrane was incubated with a horseradish peroxidase-conjugated secondary antibody (Cat. No. 7074, Cell Signaling Technology) diluted 1:3000 for CaMKII and phosphorylated CaMKII or 1:9000 for β-actin at room temperature for 1 h, immunolabeled proteins were visualized using the ECL select Western blot detection system (GE Healthcare, Tokyo, Japan) and Amersham Imager 600 (GE Healthcare). The expression level of each protein was quantified by densitometry using ImageJ. Phosphorylated CaMKII levels were normalized with CaMKII levels. Data are expressed as relative values divided by the mean of the control group.

### 2.11. Real-Time PCR

Total RNA was isolated from cells and tissue samples using an RNeasy Plus Mini Kit (Qiagen, Hilden, Germany) as previously described [33]. A reverse transcription reaction was performed using a High-Capacity cDNA Reverse Transcription Kit (Applied Biosystems, Foster, CA, USA). cDNA was subjected to SYBR Green-based real-time PCR using a Fast SYBR Green Master Mix (Applied Biosystems) and ABI PRISM 7900HT (Applied Biosystems). The conditions of the PCR were as follows: at 95°C for 20 s, followed by 40 cycles at 95°C for 3 s and 60°C for 30 s. Each PCR primer set is shown in Table S1. The specific mRNA amplification of the target was confirmed using the ΔΔCt method and normalization with β-actin levels. Data are expressed as relative values divided by the mean of the control group.

### 2.12. Cell Proliferation Assay

Cell proliferation was analyzed according to the incorporation of BrdU in 3T3-L1 cells and ADSCs as previously described [34]. 3T3-L1 cells and ADSCs from the second passage were seeded on a 96-well plate at 5 × 10^3^ cells/well and incubated with DMEM containing 10% FBS and BrdU for 24 h. The BrdU incorporation assay was performed using a BrdU Cell Proliferation Assay Kit (Exalpha Biologicals, Shirley, MA, USA). Absorbance was measured at 450/550 nm by a microplate reader (BioRad), and data are expressed as normalized values divided by the mean of the control group.

### 2.13. Oil Red O Staining

Oil Red O staining was performed to assess lipid accumulation in 3T3-L1 cells. 3T3-L1 cells were seeded at 5.0 × 10^3^ cells/cm^2^ and cultured with DMEM containing 10% FBS, 10 μg/mL insulin, 1 nM dexamethasone, and 0.5 mM 3-isobutyl-1-methylxanthine for 12 days to induce adipogenic differentiation. Cells were fixed with 10% formalin at room temperature for 10 min and stained with 0.3% of Oil Red O in 60% isopropyl alcohol for 20 min (Muto Pure Chemicals. Tokyo, Japan). Stained cells were dissolved in isopropyl alcohol (Wako Pure Chemicals), and absorbance was read at 510 nm by the Multiskan Go microplate spectrophotometer (Thermo Fisher Scientific). Data are expressed as normalized values divided by the mean of the control group.

### 2.14. Cell Viability Assay

Trypan blue staining was used to quantify viable cells. In brief, 3T3-L1 cells and ADSCs from the second passage were seeded on a 96-well plate at 1 × 10^4^ cells/well and cultured with or without KN-93 or acremomannolipin A for 24 h. The culture medium and cells were harvested, and a drop of the cell suspension was mixed with a Trypan blue solution. The ratio of each volume was 1:1. Viable and nonviable cell numbers were counted under a light microscope, and data were expressed as the percentage of viable cells in all cells.

### 2.15. Statistical Analysis

The sample size for each experiment was selected using G∗Power 3.1 software [35] in consideration of sample and effect sizes in our previous studies [28, 36, 37]. Sample sizes for in vivo and in vitro experiments in the present study were calculated using the following parameters based on our previous studies: power, 0.8; *p* value, 0.05; tails, two. We planned sample sizes in each experiment to avoid reducing the power of the study.

Data are represented as the mean ± standard error of the mean (SEM). A two-way ANOVA followed by the Tukey–Kramer test was performed for multiple comparisons. The Mann–Whitney *U* test was used for comparisons of two groups. Differences among experimental groups were considered to be significant when *p* values were less than 0.05. To identify and eliminate outliers in the analysis of plasma creatinine levels, we used the Smirnov–Grubbs test. Statistical analyses were performed using GraphPad PRISM 7.00 software (GraphPad Software, La Jolla, CA, USA).

## 3. Results

### 3.1. Effects of KN-93 and Acremomannolipin A on Fat, Muscle, and Bone Masses in HFD-Induced Obese Mice

**Figure 1:**
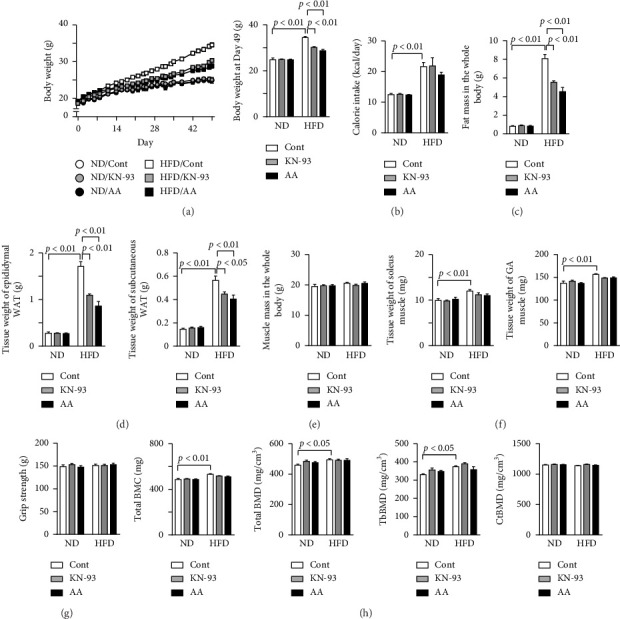
Effects of KN-93 and acremomannolipin A on HFD-induced obesity in mice. (a) KN-93 and acremomannolipin A (AA) were administered to mice fed a normal diet (ND) or high-fat diet (HFD) at doses of 5.33 and 15 μmol/kg, respectively, every other day for 8 weeks. Data on the body weights of and calorie intakes by mice fed ND or HFD. Body weight was measured every 3 or 4 days (left). Data on the body weights of mice 49 days after ND or HFD feeding were started (right). (b) Data on calorie intakes by mice fed ND or HFD. Food intake was measured for 3 days on days 54–56 after ND or HFD feeding was started and is shown as a representative of the average daily calorie intake. (c, e) Fat mass (c) and muscle mass (e) in the whole body were assessed by QCT in mice treated with or without KN-93 or AA 8 weeks after ND or HFD feeding was started. (d, f) The tissue weights of epididymal and subcutaneous WAT (d) as well as the soleus and gastrocnemius (GA) muscles (f) were measured in mice treated with or without KN-93 or AA 8 weeks after ND or HFD feeding was started. (g) The grip strengths of the four limbs were measured using a grip strength meter in mice treated with or without KN-93 or AA 8 weeks after ND or HFD feeding was started. (h) Total bone mineral content (BMC), total bone mineral density (BMD), trabecular BMD (TbBMD), and cortical BMD (CtBMD) at the tibia were assessed by QCT in mice treated with or without KN-93 or AA 8 weeks after ND or HFD feeding was started. Data represent the mean ± SEM. *n* = 8 mice in each group (a–h). Error bars of graphs indicate standard errors. Cont; Control.

The administration of both KN-93 and acremomannolipin A significantly reduced HFD-induced increases in body weight, but not calorie intake (Figures 1(a), 1(b), Table S2). In comparison with HFD-fed mice, KN-93 and acremomannolipin A induced weight losses of 12.3% and 15.4%, respectively (Figure 1(a), Table S2). The administration of both KN-93 and acremomannolipin A significantly decreased fat mass in the whole body and epididymal and subcutaneous WAT weights in HFD-fed mice (Figures 1(c), 1(d), Table S2). KN-93 and acremomannolipin A did not affect muscle mass in the whole body, soleus and gastrocnemius muscle weights, or grip strength in mice, while HFD increased soleus and gastrocnemius muscle weights (Figures 1(e), 1(f), 1(g), Table S2). Furthermore, KN-93 and acremomannolipin A did not affect BMC in the whole body, total BMD, trabecular BMD, or cortical BMD in the tibia of mice, whereas HFD increased BMC in the whole body, total BMD, and trabecular BMD (Figure 1(h), Table S2). KN-93 and acremomannolipin A did not affect the plasma levels of creatinine, AST, or ALT, erythrocyte, leukocyte, or platelet numbers, or the hemoglobin level or hematocrit in mice (Table 1).

### 3.2. Effects of KN-93 and Acremomannolipin A on WAT in HFD-Induced Obese Mice

**Figure 2:**
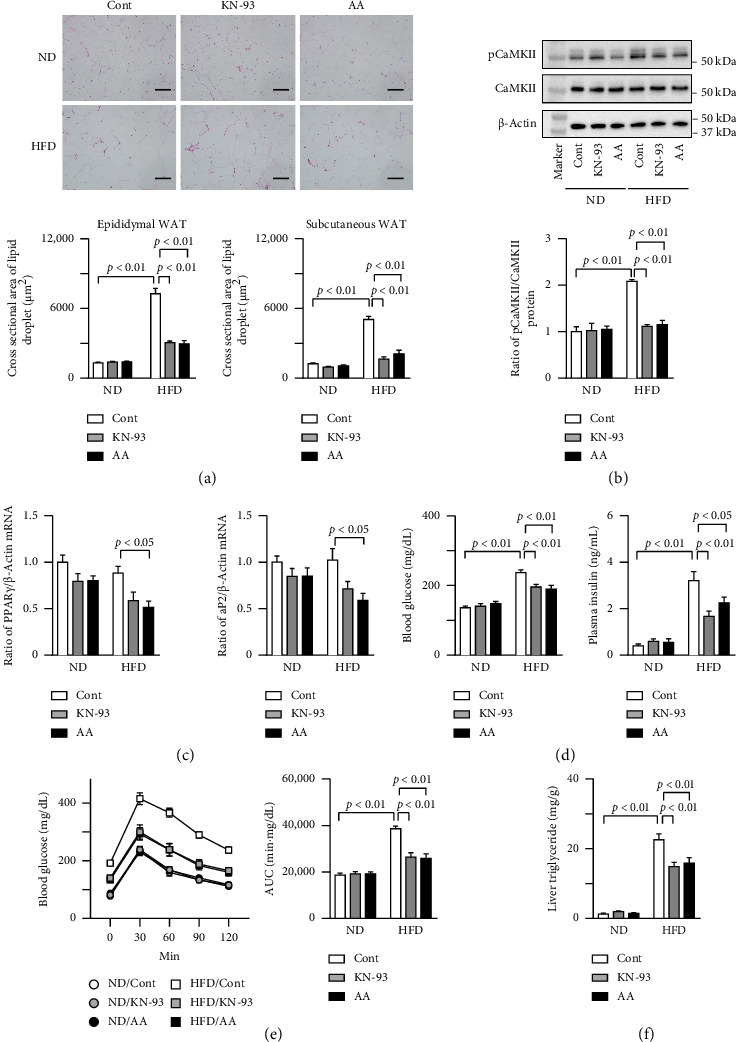
Effects of KN-93 and acremomannolipin A on HFD-induced lipid accumulation and glucose intolerance in mice. (a) KN-93 and acremomannolipin A (AA) were administered to mice fed ND or HFD at doses of 5.33 and 15 μmol/kg, respectively, every other day for 8 weeks. Representative microphotographs of hematoxylin and eosin-stained sections of the epididymal WAT of mice treated with or without KN-93 or AA 8 weeks after ND or HFD feeding was started. Scale bars indicate 100 μm. Quantification of the cross-sectional area of lipid droplets in the epididymal and subcutaneous WAT of mice 8 weeks after ND or HFD feeding was started. (b) Total proteins were extracted from the epididymal WAT of mice treated with or without KN-93 or AA 8 weeks after ND or HFD feeding was started. Western blot analyses of phosphorylated CaMKII (pCaMKII), CaMKII, and β-actin were performed. Images represent experiments performed independently at least four times. The results of Western blot analyses of pCaMKII and CaMKII were quantified and expressed as a ratio of pCaMKII to CaMKII protein levels. (c) Total RNA was extracted from the epididymal WAT of mice treated with or without KN-93 or AA 8 weeks after ND or HFD feeding was started. Real-time PCR analyses of PPARγ, aP2, and β-actin were performed. Data are expressed as a ratio to the control group. (d) Blood glucose and plasma insulin levels were measured in mice treated with or without KN-93 or AA 8 weeks after ND or HFD feeding was started. (e) Response of blood glucose to a single intraperitoneal injection of glucose in mice treated with or without KN-93 or AA 8 weeks after ND or HFD feeding was started. The area under the curve (AUC) for 120 min in the glucose tolerance test is shown. (f) Lipids were extracted from the liver tissues of mice treated with or without KN-93 or AA 8 weeks after ND or HFD feeding was started. Triglyceride levels were analyzed as described in the Materials and Methods section and are expressed as triglyceride weight per tissue weight of liver (mg/g). Data represent the mean ± SEM. *n* = 8 (a, c-f) and 4 (b) mice in each group. Error bars of graphs indicate standard errors.

The administration of both KN-93 and acremomannolipin A significantly reduced the cross-sectional areas of lipid droplets in the epididymal and subcutaneous WAT of HFD-fed mice (Figure 2(a), Table S3). KN-93 and acremomannolipin A both suppressed the phosphorylation of CaMKII in the epididymal WAT of HFD-fed mice (Figure 2(b), Table S3). Acremomannolipin A decreased PPARγ and aP2 mRNA levels in the epididymal WAT of HFD-fed mice, whereas KN-93 seemed to reduce their levels without any statistically significant differences (Figure 2(c), Table S3). Since an obese state affects glucose metabolism, we examined the effects of KN-93 and acremomannolipin A on glucose intolerance in HFD-induced obese mice. The administration of both KN-93 and acremomannolipin A significantly decreased blood glucose and insulin levels in HFD-fed mice (Figure 2(d), Table S3). Moreover, they ameliorated glucose intolerance in HFD-fed mice (Figure 2(e), Table S3). KN-93 and acremomannolipin A also significantly decreased triglyceride levels in the livers of HFD-fed mice (Figure 2(f), Table S3).

### 3.3. Effects of KN-93 and Acremomannolipin A on the Adipogenic Differentiation of 3T3-L1 and ADSCs

**Figure 3:**
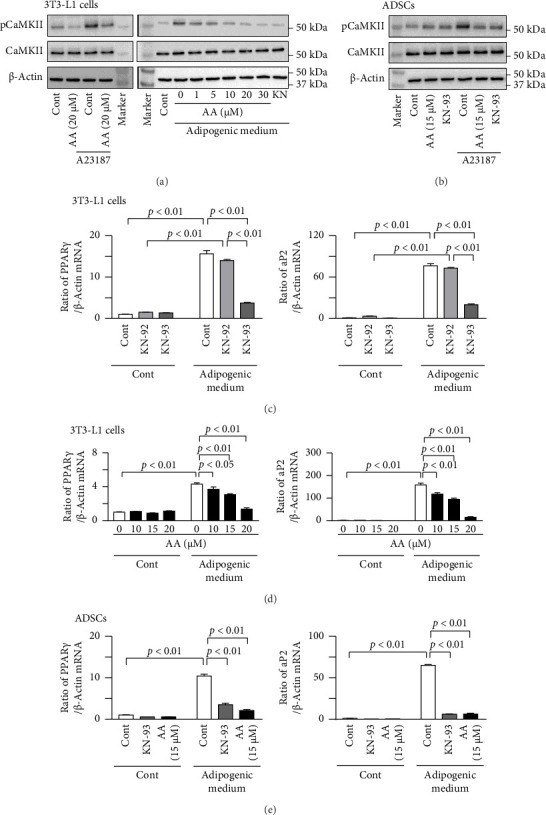
Effects of KN-93 and acremomannolipin A on the phosphorylation of CaMKII and adipogenic differentiation in 3T3-L1 cells and ADSCs. (a) 3T3-L1 cells were pretreated with or without 20 μM acremomannolipin A (AA) for 6 h, and cells were then cultured further with or without 5 μM A23187 for 1 h in the absence or presence of AA (left). 3T3-L1 cells were pretreated with the indicated concentrations of AA or 10 μM KN-93 (KN) for 6 h, and cells were then cultured further with or without the adipogenic medium containing 10% FBS, 10 μg/mL insulin, 1 nM dexamethasone, and 0.5 mM 3-isobutyl-1-methylxanthine for 1 h in the presence of AA or KN-93 (right). Total proteins were extracted from 3T3-L1 cells, and Western blot analyses of phosphorylated CaMKII (pCaMKII), CaMKII, and β-actin were performed. Images represent experiments performed independently at least four times. (b) Mouse ADSCs were pretreated with 15 μM AA or 10 μM KN-93 for 6 h, and cells were then cultured further with or without 5 μM A23187 for 1 h in the absence or presence of AA or KN-93. Total proteins were extracted from ADSCs, and Western blot analyses of phosphorylated CaMKII (pCaMKII), CaMKII, and β-actin were performed. Images represent experiments performed independently at least four times. (c) 3T3-L1 cells were cultured with or without the adipogenic medium for 6 days in the absence or presence of 10 μM KN-92 or 10 μM KN-93. Total RNA was then extracted from 3T3-L1 cells, and a real-time PCR analysis of PPARγ, aP2, or β-actin was performed. Data are expressed as a ratio to the control group. (d) 3T3-L1 cells were cultured with or without the adipogenic medium for 6 days in the presence of the indicated concentrations of AA. Total RNA was then extracted from 3T3-L1 cells, and a real-time PCR analysis of PPARγ, aP2, or β-actin was performed. Data are expressed as a ratio to the control group. (e) Mouse ADSCs were cultured with or without the adipogenic medium for 6 days in the absence or presence of 10 μM KN-93 or 15 μM AA. Total RNA was then extracted from ADSCs, and a real-time PCR analysis of PPARγ, aP2, or β-actin was performed. Data are expressed as a ratio to the control group. Data represent the mean ± SEM. *n* = 6 (c), 5 (d), and 4 (e) in each group. Error bars of graphs indicate standard errors.

Acremomannolipin A decreased the phosphorylation of CaMKII enhanced by the calcium ionophore A23187 in mouse preadipocyte 3T3-L1 cells (Figure 3(a)). The inhibitory effects of acremomannolipin A on the phosphorylation of CaMKII enhanced by the adipogenic medium were observed at concentrations > 20 μM in 3T3-L1 cells (Figure 3(a)). Moreover, acremomannolipin A at 15 μM suppressed the A23187-enhanced phosphorylation of CaMKII in mouse ADSCs (Figure 3(b)). KN-93, but not KN-92, significantly reduced the increases in PPARγ and aP2 mRNA levels induced by the adipogenic medium in 3T3-L1 cells (Figure 3(c), Table S4). Acremomannolipin A dose-dependently reduced the increases in PPARγ and aP2 mRNA levels induced by the adipogenic medium in 3T3-L1 cells (Figure 3(d), Table S5). KN-93 and acremomannolipin A both significantly reduced the increases in PPARγ and aP2 mRNA levels induced by the adipogenic medium in ADSCs (Figure 3(e), Table S6).

### 3.4. Effects of KN-93 and Acremomannolipin A on the Proliferation of and Lipid Accumulation in 3T3-L1 Cells and ADSCs

**Figure 4:**
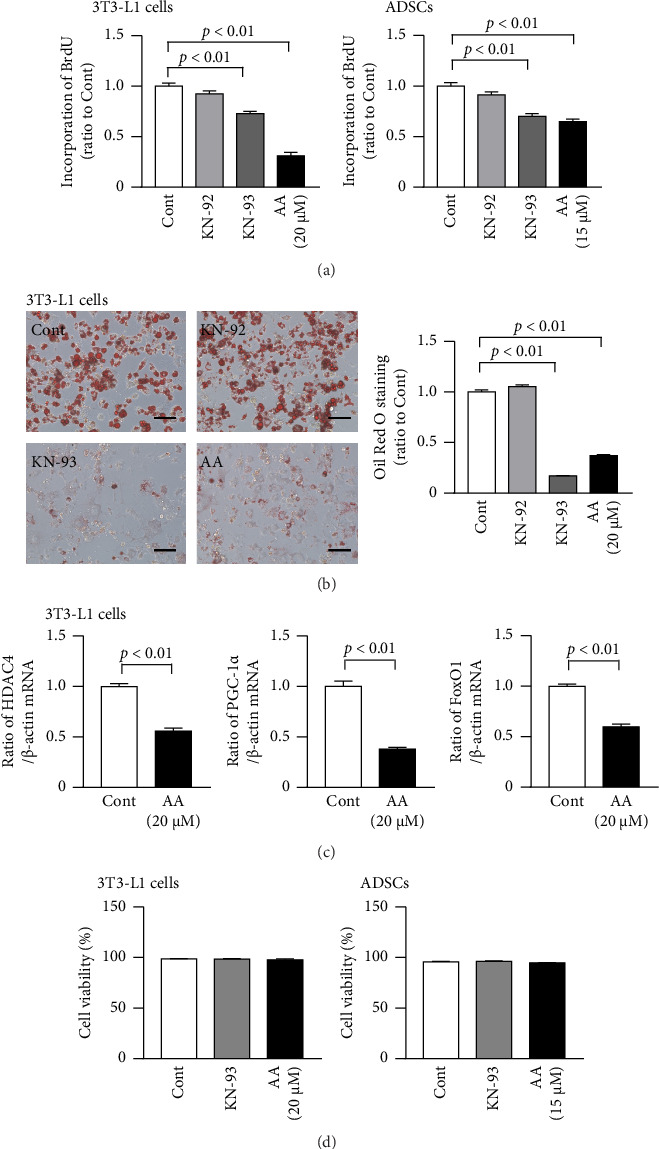
Effects of KN-93 and acremomannolipin A on cell proliferation and lipid accumulation in 3T3-L1 cells and ADSCs. (a) 3T3-L1 cells and mouse ADSCs were cultured with BrdU for 24 h in the presence or absence of 10 μM KN-92, 10 μM KN-93, or the indicated concentrations of acremomannolipin A (AA). A BrdU cell proliferation assay was then performed. Data are expressed as a ratio to the control group. (b) 3T3-L1 cells were cultured with the adipogenic medium for 12 days in the presence or absence of 10 μM KN-92, 10 μM KN-93, or 20 μM AA. Oil Red O staining was then performed. Representative microphotographs of Oil Red O–stained cells (left). Scale bars indicate 100 μm. Stained cells were quantified as described in the Methods (right). Data are expressed as a ratio to the control group. (c) Total RNA was extracted from 3T3-L1 cells cultured with the adipogenic medium for 6 days in the presence or absence of 20 μM AA. A real-time PCR analysis of HDAC4, PGC-1*α*, FoxO1, or β-actin was performed. Data are expressed as a ratio to the control group. (d) 3T3-L1 cells and mouse ADSCs were treated with or without 10 μM KN-93 or the indicated concentration of AA for 24 h. A cell viability assay was performed as described in the Materials and Methods section. Data are expressed as the percentage of viable cells in all cells. Data represent the mean ± SEM. *n* = 6 (a, b), 5 (c), 8 (d, left), and 4 (d, right) in each group. Error bars of graphs indicate standard errors.

KN-93 and acremomannolipin A both significantly suppressed proliferation assessed by the incorporation of BrdU in 3T3-L1 cells and mouse ADSCs (Figure 4(a), Table S7). Moreover, KN-93 and acremomannolipin A both significantly attenuated enhancements in lipid accumulation induced by the adipogenic medium in 3T3-L1 cells (Figure 4(b), Table S7). We then examined the effects of acremomannolipin A on the expression of downstream molecules in the CaMKII pathway, such as histone deacetylase (HDAC) 4, PPARγ coactivator-1*α* (PGC-1*α*), and FoxO1, in 3T3-L1 cells. Acremomannolipin A decreased HDAC4, PGC-1*α*, and FoxO1 mRNA levels in 3T3-L1 cells cultured in the adipogenic medium for 6 days (Figure 4(c), Table S7). However, KN-93 and acremomannolipin A did not affect the viability of 3T3-L1 cells or ADSCs (Figure 4(d), Table S7).

## 4. Discussion

The present results revealed that two CaMKII inhibitors, KN-93 and acremomannolipin A, decreased fat mass, attenuated HFD-induced increases in epididymal and subcutaneous WAT weights, and ameliorated glucose intolerance without any obvious side effects in mice. Furthermore, these CaMKII inhibitors reduced adipogenic differentiation, cell proliferation, and lipid accumulation in 3T3-L1 cells and ADSCs.

Excess energy in the body is stored in WAT, which is followed by an increase in fat mass due to the hypertrophy and hyperplasia of adipocytes [9, 35]. Previous studies suggested that intracellular calcium was crucial for the regulation of adipogenesis in human and mouse adipocytic cells [36, 37]. Among calcium signaling molecules, CaMKII has been shown to contribute to adipogenic differentiation, Fas-induced lipolysis, the insulin-induced translocation of GLUT4, and the enhancement of angiotensin II–induced insulin resistance in 3T3-L1 cells [17, 38–40]. These findings suggest that calcium/CaMKII signaling plays crucial roles in adipocyte biology and has potential as a target for the treatment of obesity. In the present study, the administration of two CaMKII inhibitors, KN-93 and acremomannolipin A, significantly decreased body weight gain, fat mass in the whole body, and HFD-induced increases in epididymal and subcutaneous WAT weights in mice without any changes in calorie intake, renal or hepatic indices, or blood cell counts. Moreover, KN-93 and acremomannolipin A did not affect soleus and gastrocnemius muscle weights, total muscle masses, or cortical and trabecular BMD in HFD-fed mice. These results indicate that the CaMKII inhibitors, KN-93 and acremomannolipin A, attenuated diet-induced obesity in mice through a decrease in fat mass.

Accumulating evidence indicates that CaMKII plays several roles in adipocytes [38–40]. In the present study, we identified acremomannolipin A as a novel inhibitor of CaMKII based on the phosphorylation of CaMKII in Western blots. We also demonstrated that KN-93 and acremomannolipin A both decreased the expression of PPARγ and aP2 in 3T3-L1 cells and ADSCs, which is consistent with previous findings, showing that the temporal activation of CaMKII was necessary for the adipogenic differentiation of 3T3-L1 cells [17]. Collectively, these findings and the present results suggest that the inhibition of calcium/CaMKII signaling suppressed adipogenic differentiation in mesenchymal stem cells and preadipocytes. On the other hand, the number and size of adipocytes both increase during the development of obesity in rodents [9]. We showed that KN-93 and acremomannolipin A both significantly suppressed the incorporation of BrdU in 3T3-L1 and ADSCs. Moreover, KN-93 and acremomannolipin A both significantly decreased lipid accumulation in 3T3-L1 cells and the size of lipid droplets in epididymal WAT of HFD-fed mice. These results indicate that CaMKII inhibitors attenuated HFD-induced obesity presumably by suppressing adipogenic differentiation, proliferation, and lipid accumulation in adipocytes. Rapold et al. showed that the Fas ligand promoted lipolysis by activating CaMKII in 3T3-L1 cells [38]. Therefore, we speculated that a decrease in lipid accumulation in adipocytes following the administration of CaMKII inhibitors in mice may be due to decreases in adipogenic differentiation and proliferation rather than the facilitation of lipolysis in adipocytes.

Previous studies revealed the involvement of CaMKII in glucose metabolism in mice [13, 14, 39, 40]. The present study showed that the administration of KN-93 and acremomannolipin A significantly decreased blood levels of glucose and insulin and ameliorated glucose intolerance in HFD-fed mice. These results suggest that CaMKII inhibitors improve abnormalities in glucose metabolism induced by the obese state in mice. Yip et al. demonstrated that CaMKII played a role in the insulin-induced translocation of GLUT4 in adipocytes [40]. Gao et al. revealed that redox signal-mediated TRPM2 facilitated angiotensin II–induced insulin resistance via CaMKII activation in 3T3-L1 cells [39]. Moreover, Dai et al. found that an adipocyte-specific CaMKII deficiency attenuated glucose intolerance in obese mice [16]. Taken together, these findings and the present results suggest that CaMKII inhibitors effectively ameliorated glucose metabolism abnormalities in mice, although further studies are needed to clarify the effects of CaMKII inhibitors on glucose metabolism in humans. Since previous studies using liver-specific CaMKII-deleted mice showed that CaMKII mediated the suppression of insulin signaling and increases in glucose neogenesis in the livers of obese mice [13, 14], we cannot rule out the possibility that KN-93 and acremomannolipin A attenuated glucose intolerance by inhibiting CaMKII in the livers of obese mice.

Obesity is related to various disorders, such as diabetes, cardiovascular diseases, hyperlipidemia, and the exacerbation of COVID-19 [41–43]. Although the treatment of obesity is crucial for the extension of a healthy lifespan, there are limitations to the efficacy of conventional medication for obesity. Yanovski and Yanovski revealed that most patients with obesity expected a body weight reduction > 15% by interventions [3]. However, bariatric surgery is currently the only approach that achieves sufficient weight loss in patients with obesity. We herein showed that the CaMKII inhibitors, KN-93 and acremomannolipin A, ameliorated the development of diet-induced obesity without any significant health issues examined in mice. Dai et al. revealed that an adipocyte CaMKII deficiency attenuated obesity-associated glucose intolerance and TNF-α-induced inflammation in mice [16]. Therefore, CaMKII inhibitors have potential in the treatment of obesity and its associated disorders. The present results demonstrate that the CaMKII inhibitors examined did not affect bone parameters in mice with or without HFD. A previous study revealed that CaMKII deficiency in osteoblast lineage cells decreased bone mass in mice [44]. Therefore, we cannot exclude the possibility that the administration of CaMKII inhibitors at high doses may reduce bone mass by suppressing osteoblastic bone formation.

In the present study, we used the glycolipid acremomannolipin A, which inhibited CaMKII/IV activity in preliminary experiments (data not shown). Acremomannolipin A antagonized the phosphorylation of CaMKII enhanced by a calcium ionophore or the adipogenic medium and decreased the expression of downstream molecules in the CaMKII signaling pathway, such as HDAC4, PGC-1*α*, and FoxO1, in 3T3-L1 cells. These results indicate that acremomannolipin A is an inhibitor of CaMKII signaling in adipose tissues. Further in vitro studies are needed to characterize the specificity of acremomannolipin A and the molecular mechanisms underlying the inhibition of CaMKII.

There is a limitation in this study. Since CaMKII inhibitors examined were only administered to mice for 8 weeks, therefore, we cannot exclude the possibility that their administration for a longer period may induce unexpected side effects in vivo.

We initially examined the effects of mechanical stress or exercise on skeletal muscle and bone with or without obesity in mice [45]. Then, we performed the experiments using several pathway inhibitors, including calmodulin kinase inhibitors, for the mechanistic studies. However, the present study suggested that calmodulin kinase inhibitors do not affect the parameters of muscle and bone modulated by obesity in mice.

## 5. Conclusions

We herein demonstrated that the CaMKII inhibitors KN-93 and acremomannolipin A prevented the development of diet-induced obesity in mice presumably through the suppression of adipogenic differentiation, cell proliferation, and lipid accumulation in adipocytes. The present results suggest the potential of KN-93 and acremomannolipin A as antiobesity drugs.

## Figures and Tables

**Table 1:** Effects of KN-93 and acremomannolipin A on the levels of plasma creatinine, aspartate aminotransferase, alanine aminotransferase, hemoglobin, and hematocrit as well as blood cell count in mice.

	ND/Cont	ND/KN-93	ND/AA	HFD/Cont	HFD/KN-93	HFD/AA
Cr (mg/dL) (CI)	0.219 ± 0.011 (0.193–0.245)	0.231 ± 0.015 (0.196–0.267)	0.293 ± 0.052 (0.171–0.415)	0.290 ± 0.021 (0.238–0.342)	0.318 ± 0.044 (0.215–0.422)	0.303 ± 0.060 (0.161–0.445)
AST (IU/L) (CI)	22.8 ± 3.2 (15.18–30.43)	18.7 ± 1.9 (14.28–23.16)	23.7 ± 2.9 (16.89–30.52)	25.4 ± 1.8 (21.19–29.52)	20.8 ± 1.2 (17.98–23.69)	28.0 ± 2.2 (22.73–33.21)
ALT (IU/L) (CI)	11.0 ± 0.44 (10.00–12.06)	12.0 ± 0.70 (10.37–13.66)	11.4 ± 0.18 (10.92–11.78)	11.1 ± 0.25 (10.56–11.72)	10.6 ± 0.17 (10.16–10.95)	10.9 ± 0.26 (10.28–11.51)
RBC (10^4^/μL) (CI)	761 ± 14 (727.3–794.7)	770 ± 9 (748.3–791.5)	778 ± 11 (751.0–804.3)	817 ± 19 (773.4–861.3)	782 ± 21 (733.7–831.1)	761 ± 32 (684.0–837.0)
WBC (10^2^//μl) (CI)	12.6 ± 1.3 (9.47–15.65)	16.6 ± 2.2 (11.55–21.73)	13.7 ± 1.7 (9.64–17.66)	14.1 ± 1.5 (10.56–17.54)	15.3 ± 2.0 (10.44–20.11)	14.8 ± 1.5 (11.28–18.40)
PLT (10^4^/μL) (CI)	101 ± 14 (68.2–133.8)	121 ± 5 (109.8–133.1)	130 ± 2 (124.5–135.2)	106 ± 4 (97.3–114.9)	108 ± 4 (99.2–117.2)	109 ± 6 (94.3–123.5)
Hb (g/dL) (CI)	11.5 ± 0.2 (11.01–12.01)	11.5 ± 0.1 (11.20–11.83)	11.5 ± 0.2 (11.04–11.91)	12.0 ± 0.2 (11.51–12.46)	11.6 ± 0.3 (10.97–12.16)	11.2 ± 0.5 (10.07–12.23)
Ht (%) (CI)	38.2 ± 0.7 (36.63–39.79)	38.3 ± 0.6 (36.90–39.68)	37.7 ± 0.5 (36.50–38.88)	40.5 ± 0.7 (38.74–42.23)	39.5 ± 0.5 (38.25–40.65)	38.1 ± 1.6 (34.37–41.78)

*Note:* Plasma samples were collected from mice fed normal diet or HFD for 8 weeks. Levels of plasma creatinine (Cr), aspartate aminotransferase (AST), alanine aminotransferase (ALT), hemoglobin (Hb), and hematocrit (Ht) as well as number of erythrocytes (RBC), leukocytes (WBC), and platelets (PLT) were analyzed.

Abbreviations: AA, acremomannolipin A; CI, confidence intervals; ND, normal diet.

## Data Availability

The data that support the findings of this study are available from the corresponding author upon reasonable request.

## Supporting Information

Supporting Information Supporting Table S1. Primers used in real-time PCR experiments.

Supporting Information Supporting Table S2. Confidence intervals of data shown in Figure 1.

Supporting Information Supporting Table S3. Confidence intervals of data shown in Figure 2.

Supporting Information Supporting Table S4. Confidence intervals of data shown in Figure 3(c).

Supporting Information Supporting Table S5. Confidence intervals of data shown in Figure 3(d).

Supporting Information Supporting Table S6. Confidence intervals of data shown in Figure 3(e).

Supporting Information Supporting Table S7. Confidence intervals of data shown in Figure 4.
