# Supplementary material for: Calcium/Calmodulin-Dependent Protein Kinase II Inhibitors Mitigate High-Fat Diet–Induced Obesity in Mice
Source: J Obes. 2025 Jun 30;2025:5530467. doi: 10.1155/jobe/5530467 (PMC12259312; doi:10.1155/jobe/5530467)
Supplement: Supporting Information — Supporting Table S1. Primers used in real-time PCR experiments. [file 5530467.f1.docx]

**Table S1.** Primers used in real-time PCR experiments.

| Gene |  | Primer sequence |
| --- | --- | --- |
| PPARγ  aP2  HDAC4  PGC-1α  FoxO1 | Forward  Reverse  Forward  Reverse  Forward  Reverse  Forward  Reverse  Forward  Reverse | 5’-GGAAAGACAACGGACAAATCAC-3’  5’-TACGGATCGAAACTGGCAC-3’  5’-ATCACCGCAGACGACAGGA-3’  5’-CTCATGCCCTTTCATAAACT-3’  5’-GGCGAGCACAGAGGTGAAGATG-3’  5’-GCTGTGCTGTGTCTTCCCATAC-3’  5’-CCCTGCCATTGTTAAGACC-3’  5’-TGCTGCTGTTCCTGTTTTC-3’  5’-CAAAGTACACATACGGCCAATCC-3’  5’-CGTAACTTGATTTGCTGTCCTGAA-3’ |
| β-Actin | Forward  Reverse | 5’-TACCACAGGCATTGTGATGG-3’  5’-TTTGATGTCACGCACGATTT-3’ |

PPARγ, proliferator-activated receptor γ; HDAC4, histone deacetylase 4; PGC-1α, peroxisome proliferator-activated receptor γ coactivator-1α.
