# Supplementary material for: Calcium/Calmodulin-Dependent Protein Kinase II Inhibitors Mitigate High-Fat Diet–Induced Obesity in Mice
Source: J Obes. 2025 Jun 30;2025:5530467. doi: 10.1155/jobe/5530467 (PMC12259312; doi:10.1155/jobe/5530467)
Supplement: Supporting Information — Supporting Table S2. Confidence intervals of data shown in Figure 1. [file 5530467.f2.docx]

**Table S2.** Confidence intervals of data shown in Fig. 1.

|  | ND/Cont | ND/KN-93 | ND/AA | HFD/Cont | HFD/KN-93 | HFD/AA |
| --- | --- | --- | --- | --- | --- | --- |
| Body weight at Day 49 (g) | 22.97-26.61 | 24.45-25.45 | 24.14-25.44 | 33.12-35.71 | 29.39-30.99 | 27.27-30.13 |
| Calorie intake  (kcal/day) | 11.61-13.37 | 12.00-13.23 | 11.85-12.85 | 18.50-24.78 | 15.53-28.17 | 16.80-21.02 |
| Fat mass in the whole body (g) | 0.614-1.009 | 0.829-1.056 | 0.650-1.014 | 6.965-9.157 | 5.121-5.927 | 3.487-5.576 |
| Tissue weight of epididymal WAT (g) | 0.216-0.339 | 0.252-0.302 | 0.235-0.307 | 1.472-1.950 | 1.021-8.736 | 0.625-1.094 |
| Tissue weight of subcutaneous WAT (g) | 0.125-0.1666 | 0.135-0.175 | 0.131-0.187 | 0.478-0.650 | 0.406-0.489 | 0.321-0.486 |
| Muscle mass in the whole body (g) | 17.72-21.20 | 18.94-20.50 | 18.92-20.60 | 20.03-21.12 | 19.01-20.70 | 19.26-21.73 |
| Tissue weight of soleus muscle (mg) | 8.95-10.92 | 9.32-10.31 | 9.19-11.19 | 11.26-12.74 | 10.14-12.23 | 10.13-11.87 |
| Tissue weight of GA muscle (mg) | 125.1-148.9 | 135.6-146.9 | 131.8-141.4 | 152.3-160.2 | 145.5-151.0 | 143.9-153.5 |
| Grip strength | 139.5-157.7 | 146.0-159.8 | 139.8-155.1 | 144.4-157.6 | 145.0-156.9 | 146.0-160.1 |
| Total BMC (mg) | 467.5-504.9 | 481.5-499.4 | 469.1-501.5 | 516.9-545.3 | 506.4-525.6 | 492.2-525.1 |
| Total BMD (mg/cm^3^) | 442.6-474.3 | 467.4-501.4 | 457.8-492.5 | 475.1-511.2 | 474.0-507.3 | 464.7-516.5 |
| TbBMD (mg/cm^3^) | 318.0-342.0 | 330.5-380.7 | 335.1-359.9 | 362.7-385.2 | 376.5-402.8 | 316.6-397.6 |
| CtBMD (mg/cm^3^) | 1130-1169 | 1144-1174 | 1144-1168 | 1125-1151 | 1139-1174 | 1130-1160 |

ND; normal diet, HFD; high fat diet, AA; acremomannolipin A, WAT; white adipose tissue, GA; gastrocnemius, BMC; bone mineral content, TbBMD; trabecular bone mineral density, CtBMD; cortical bone mineral density.
