# Supplementary material for: Calcium/Calmodulin-Dependent Protein Kinase II Inhibitors Mitigate High-Fat Diet–Induced Obesity in Mice
Source: J Obes. 2025 Jun 30;2025:5530467. doi: 10.1155/jobe/5530467 (PMC12259312; doi:10.1155/jobe/5530467)
Supplement: Supporting Information — Supporting Table S3. Confidence intervals of data shown in Figure 2. [file 5530467.f3.docx]

**Table S3.** Confidence intervals of data shown in Fig. 2.

|  | ND/Cont | ND/KN-93 | ND/AA | HFD/Cont | HFD/KN-93 | HFD/AA |
| --- | --- | --- | --- | --- | --- | --- |
| Cross sectional area of lipid droplet in epididymal WAT (µm^2^) | 1137-1480 | 1242-1527 | 1227-1523 | 6149-8381 | 2708-3406 | 2410-3526 |
| Cross sectional area of lipid droplet in subcutaneous WAT (µm^2^) | 1104-1391 | 783.1-1115 | 850.3-1265 | 4386-5708 | 1128-2129 | 1364-2824 |
| Ratio of pCaMKII /CaMKII protein | 0.659-1.341 | 0.844-1.263 | 0.534-1.512 | 1.957-2.203 | 0.851-1.450 | 0.994-1.233 |
| Ratio of PPARγ /β-Actin mRNA | 0.821-1.179 | 0.599-0.991 | 0.680-0.923 | 0.714-1.051 | 0.372-0.803 | 0.357-0.673 |
| Ratio of aP2 /β-Actin mRNA | 0.846-1.156 | 0.641-1.052 | 0.646-1.056 | 0.734-1.313 | 0.524-0.901 | 0.413-0.767 |
| Blood glucose (mg/dl) | 125.6-147.4 | 123.9-157.6 | 132.9-157.6 | 218.8-255.4 | 178.1-213.4 | 165.7-214.8 |
| Plasma insulin (ng/ml) | 0.220-0.586 | 0.345-0.847 | 0.234-0.348 | 2.303-4.110 | 1.269-1.870 | 1.651-2.841 |
| AUC | 16877-20529 | 16937-21425 | 17224-21206 | 35818-41334 | 21964-30855 | 21334-30378 |
| Liver triglyceride (mg/g) | 0.704-1.842 | 1.410-2.495 | 1.002-1.994 | 18.60-26.61 | 12.04-17.77 | 12.20-19.59 |

ND; normal diet, HFD; high fat diet, AA; acremomannolipin A, WAT; white adipose tissue, pCaMKII; phosphorylated CaMKII, AUC; area under the curve.
