# Supplementary material for: Calcium/Calmodulin-Dependent Protein Kinase II Inhibitors Mitigate High-Fat Diet–Induced Obesity in Mice
Source: J Obes. 2025 Jun 30;2025:5530467. doi: 10.1155/jobe/5530467 (PMC12259312; doi:10.1155/jobe/5530467)
Supplement: Supporting Information — Supporting Table S4. Confidence intervals of data shown in Figure 3(c). [file 5530467.f4.docx]

**Table S4.** Confidence intervals of data shown in Fig. 3C.

|  | Cont/Cont | KN-92/Cont | KN-93/Cont | Cont/AM | KN-92/AM | KN-93/AM |
| --- | --- | --- | --- | --- | --- | --- |
| Ratio of PPARγ /β-Actin mRNA | 0.892-1.108 | 1.462-1.568 | 1.308-1.428 | 13.59-17.66 | 13.31-14.70 | 3.407-4.112 |
| Ratio of aP2 /β-Actin mRNA | 0.749-1.251 | 2.394-4.379 | 0.501-0.686 | 68.66-84.13 | 69.61-76.19 | 17.23-23.00 |

AA; acremomannolipin A, AM; adipogenic medium.
