# Supplementary material for: Calcium/Calmodulin-Dependent Protein Kinase II Inhibitors Mitigate High-Fat Diet–Induced Obesity in Mice
Source: J Obes. 2025 Jun 30;2025:5530467. doi: 10.1155/jobe/5530467 (PMC12259312; doi:10.1155/jobe/5530467)
Supplement: Supporting Information — Supporting Table S5. Confidence intervals of data shown in Figure 3(d). [file 5530467.f5.docx]

**Table S5.** Confidence intervals of data shown in Fig. 3D.

| **Cont** | 0 µM AA | 10 µM AA | 15 µM AA | 20 µM AA |
| --- | --- | --- | --- | --- |
| Ratio of PPARγ /β-Actin mRNA | 0.922-1.081 | 1.056-1.128 | 0.791-0.977 | 1.017-1.219 |
| Ratio of aP2 /β-Actin mRNA | 0.525-1.475 | 0.766-1.722 | 0.426-0.706 | 0.155-0.193 |
|  |  |  |  |  |
| **Adipogenic medium** | 0 µM AA | 10 µM AA | 15 µM AA | 20 µM AA |
| Ratio of PPARγ /β-Actin mRNA | 3.937-4.711 | 2.881-4.499 | 2.792-3.352 | 0.927-1.793 |
| Ratio of aP2 /β-Actin mRNA | 138.1-179.0 | 98.17-136.1 | 80.13-108.4 | 6.051-22.87 |

AA; acremomannolipin A.
