# Supplementary material for: Calcium/Calmodulin-Dependent Protein Kinase II Inhibitors Mitigate High-Fat Diet–Induced Obesity in Mice
Source: J Obes. 2025 Jun 30;2025:5530467. doi: 10.1155/jobe/5530467 (PMC12259312; doi:10.1155/jobe/5530467)
Supplement: Supporting Information — Supporting Table S6. Confidence intervals of data shown in Figure 3(e). [file 5530467.f6.docx]

**Table S6.** Confidence intervals of data shown in Fig. 3E.

|  | Cont/Cont | KN-93/Cont | AA/Cont | Cont/AM | KN-93/AM | AA/AM |
| --- | --- | --- | --- | --- | --- | --- |
| Ratio of PPARγ /β-Actin mRNA | 0.769-1.236 | 0.513-0.607 | 0.469-0.626 | 8.976-11.86 | 2.424-4.611 | 1.150-3.025 |
| Ratio of aP2 /β-Actin mRNA | 0.823-1.177 | 0.158-0.388 | 0.301-0.420 | 61.50-68.43 | 4.824-7.146 | 2.756-9.639 |

AA; acremomannolipin A, AM; adipogenic medium.
