# Supplementary material for: Calcium/Calmodulin-Dependent Protein Kinase II Inhibitors Mitigate High-Fat Diet–Induced Obesity in Mice
Source: J Obes. 2025 Jun 30;2025:5530467. doi: 10.1155/jobe/5530467 (PMC12259312; doi:10.1155/jobe/5530467)
Supplement: Supporting Information — Supporting Table S7. Confidence intervals of data shown in Figure 4. [file 5530467.f7.docx]

**Table S7.** Confidence intervals of data shown in Fig. 4.

|  | Cont | KN-92 | KN-93 | AA |
| --- | --- | --- | --- | --- |
| Incorporation of BrdU in 3T3-L1 cells | 0.917-1.083 | 0.853-0.999 | 0.666-0.792 | 0.215-0.405 |
| Incorporation of BrdU in ADSCs | 0.910-1.090 | 0.839-0.991 | 0.629-0.773 | 0.596-0.705 |
| Oil Red O staining | 0.947-1.053 | 1.009-1.097 | 0.168-0.175 | 0.349-0.393 |
| Ratio of HDAC4 /β -Actin mRNA | 0.912-1.084 | - | - | 0.477-0.639 |
| Ratio of PGC-1α /β -Actin mRNA | 0.858-1.142 | - | - | 0.330-0.430 |
| Ratio of FoxO1 /β -Actin mRNA | 0.936-1.060 | - | - | 0.514-0.678 |
| Cell viability in 3T3-L1 cells (%) | 98.44-99.27 | - | 97.16-99.81 | 96.24-99.51 |
| Cell viability in ADSCs (%) | 94.63-97-25 | - | 94.91-97.62 | 94.29-95.33 |

AA; acremomannolipin A.
